# Comprehensive Study on Design Optimization and Retardation Mechanism of SS-GGBS-FA Ternary Geopolymer Mortar

**DOI:** 10.3390/ma18102388

**Published:** 2025-05-20

**Authors:** Chen Jin, Jian Geng, Genjin Liu

**Affiliations:** 1 College of Civil Engineering and Architecture, Zhejiang University, Hangzhou 310017, China; jinchenguo0917@163.com; 2 School of Civil Engineering and Architecture, Ningbo Tech University, Ningbo 315100, China; liugenjin@163.com

**Keywords:** steel slag, geopolymer, barium chloride, retarding mechanism

## Abstract

A ternary geopolymer mortar (TGM) was synthesized using steel slag (SS), granulated blast furnace slag (GGBS), and fly ash (FA) as raw materials. The effect of the SS content (0–60%) and the GGBS/FA mass ratio (5:1 to 1:5) on the TGM’s setting time was studied. To address the issue of rapid setting, the impact of different mixing methods ((A) dry mixing, (B) pre-dissolution, and (C) pre-coating) and dosages of BaCl_2_ on the setting and hardening properties of TGM was further explored. The hydration product evolution and microstructural characteristics were characterized using X-ray diffraction (XRD), scanning electron microscopy (SEM), and energy dispersive spectrometry (EDS), with an in-depth analysis of the retarding mechanism of BaCl_2_. The results indicate that, as the steel slag content increases, the setting time of TGM significantly shortens. The setting time decreases slightly with an increase in the GGBS/FA mass ratio. The mixing method influences the retarding effect of BaCl_2_, with the C method showing significant advantages over both the A and B methods. Under the C mixing method, BaCl_2_ consumes the alkaline components (SiO_3_^2−^) in the alkaline activator and forms a BaSiO_3_ coating layer on the precursor surface, which further delays the hydration process of the precursor particles. This study provides a promising approach for the high-value utilization of multi-source solid waste and suggests that future research should focus on large-scale application strategies and long-term performance evaluation to support its practical use in sustainable construction.

## 1. Introduction

As the world’s largest developing country, China’s demand for ordinary Portland cement (OPC) remains high, and its production has consistently led the world for decades. However, the production of OPC is associated with several environmental issues. On the one hand, it requires the consumption of large quantities of non-renewable resources such as limestone, and, on the other hand, it consumes significant amounts of energy during the high-temperature calcination process. More critically, the OPC industry emits large amounts of CO_2_, SO_2_, etc., and other greenhouse gases and pollutants every year, causing severe environmental damage [1,2]. In contrast, geopolymers, a new type of binding material made from aluminosilicate precursors such as fly ash and slag, exhibit significant advantages in terms of both environmental impact and performance. On the one hand, compared with OPC, the production of geopolymers results in lower CO_2_ emissions and energy consumption [3], making them a more sustainable option. On the other hand, geopolymers demonstrate superior mechanical properties and durability [4,5]. Due to these advantages, geopolymers are seen as an environmentally friendly alternative to OPC and have become a major research focus in recent years both domestically and internationally.

Steel slag (SS), a byproduct of the steel industry, accounts for approximately 15–20% of crude steel production [6]. Since 2017, China’s crude steel production has exceeded half of the global total, and, in 2020, China’s crude steel production reached 1.053 billion tons, making up 56.5% of the world’s total. Based on the annual crude steel production and assuming 15% steel slag generation, China’s steel slag production in 2020 surpassed 150 million tons [7]. However, the current utilization rate of steel slag in China is only 22%, and a large amount of steel slag continues to be stockpiled and discarded [8]. The storage and landfill of steel slag not only occupy vast land resources but also pose significant risks due to the leaching of heavy metal ions. Therefore, exploring new pathways for the resource utilization of steel slag and conducting systematic feasibility and benefit assessments have become research hotspots in the field of solid waste disposal [6,9,10]. However, the resource utilization of steel slag is hindered by its complex composition, difficult grindability, and poor volume stability (e.g., containing free CaO and MgO). Notably, using a high-modulus sodium silicate-based alkali activation system to prepare geopolymers containing steel slag can promote the rapid participation of free CaO and MgO in the polymerization reaction, thereby improving the volume stability of the product [11], offering a feasible route for the high-value utilization of steel slag.

Currently, the research on geopolymers mainly focuses on binary geopolymer systems, with ternary geopolymer systems still requiring further exploration. Studies have shown that, in ternary geopolymers, the combination of multiple solid waste materials can generate synergistic effects, which significantly influence the hydration process and microstructure formation at different stages, resulting in complementary performance advantages [4,12,13]. Therefore, comprehensive research on the preparation technology of steel slag-based ternary geopolymers is essential. Such research can reveal their synergistic mechanisms and enhance overall performance, thereby providing scientific support for the development of green building materials.

Common raw materials used in geopolymers (e.g., slag and certain steel slags) are typically high-calcium active materials. Geopolymers made from high-calcium active materials often experience issues such as short setting times and rapid loss of flowability [14]. To optimize the performance of these binders, retarders are commonly added to control setting time. However, in geopolymer systems, there are relatively few additives that can effectively delay setting time, and traditional cement retarders often fail in highly alkaline environments. Li et al. [15] introduced BaCl_2_ as a retarder in ternary ultra-high-performance geopolymer concrete, finding that a 2% addition of BaCl_2_ significantly delayed the setting process, with the initial and final setting times reaching 213 and 265 min, respectively, extending by 204% and 223% compared with the control group. Therefore, BaCl_2_, as a potential high-efficiency retarder for geopolymers, warrants further exploration through systematic research and analysis. Meanwhile, Toobpeng et al. [16] investigated the effects of different sugars on high-calcium fly ash-based geopolymers using both wet and dry mixing methods. The results showed that, compared with the dry mixing process, the wet mixing process significantly reduced the heat release rate of the fresh slurry and extended both the initial and final setting times. This indicates that the mixing method of retarders can also influence the retarding effect of geopolymers. However, the current research on mixing methods is insufficient, and experimental approaches vary. Therefore, it is necessary to explore the effects of the same retarder on the setting time of geopolymers under different mixing methods.

This study aims to develop a ternary geopolymer mortar using steel slag, ground granulated blast furnace slag (GGBS), and fly ash (FA). The effects of steel slag content and the mass ratio of GGBS to FA on the setting time and compressive strength of the geopolymer mortar were investigated. Based on the optimized mix proportions, further research was conducted to evaluate the effects of the BaCl_2_ mixing method and dosage on the setting time, workability, heat evolution, phase composition, and microstructure of the geopolymer mortar. Additionally, the retarding mechanism model of BaCl_2_ was also established to provide theoretical support for the practical application of SS–GGBS–FA ternary geopolymers in engineering projects.

## 2. Raw Materials and Experiment Methods

### 2.1. Raw Materials

**Figure 1:**
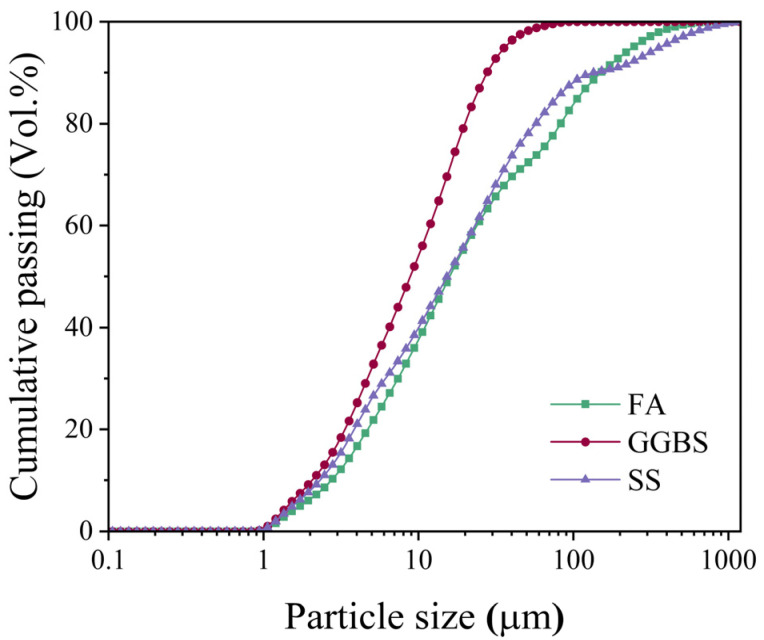
The particle size distribution of raw materials.

The raw materials utilized in this study included Grade S95 ground granulated blast furnace slag (GGBS), Class F fly ash (FA), and steel slag (SS) powder, all sourced from Ningbo, China, with respective specific surface areas of 286 m^2^/kg, 410 m^2^/kg, and 325 m^2^/kg as measured by the Blaine air permeability method. Chemical composition analysis conducted via X-ray fluorescence spectroscopy (XRF) revealed the oxide constituents presented in Table 1, while particle size distribution characteristics determined by BT-2002 laser diffraction analysis are illustrated in Figure 1.

The alkaline activation system was formulated using industrial-grade sodium silicate solution (water glass) with a modulus of 2.25, 50° Bé density, and composition of 13.75% Na_2_O and 29.99% SiO_2_, modified with analytical-grade sodium hydroxide (≥96.0% purity) for modulus adjustment. Analytical-grade barium chloride dihydrate (BaCl_2_·2H_2_O) in white crystalline form was utilized as the retarder.

Table 1 shows the alkalinity coefficient of the steel slag, M = (CaO)/(SiO_2_ + P_2_O_5_) = 2.97 > 2.50, indicating that it is a high-alkalinity steel slag.

### 2.2. Mix Design

The experimental design maintained a fixed 1:1 mass ratio between GGBS and FA to form the binary precursor system. To investigate the influence of SS content, progressive replacement of the binary precursor was conducted at 0%, 20%, 30%, 40%, 50%, and 60% by mass. For ternary geopolymer evaluation, the SS content was fixed at 40% while varying the GGBS/FA mass ratio across four proportions (1:5, 1:2, 2:1, and 5:1) to systematically examine its effect on setting characteristics. The specific proportions of the mixtures are given in Table 2.

### 2.3. Geopolymer Preparation

#### 2.3.1. Alkaline Activator Preparation

Flake sodium hydroxide (NaOH) was introduced into sodium silicate solution (original modulus = 2.25) to adjust its modulus to 1.5, yielding a modified sodium silicate solution. This solution was then diluted with deionized water to achieve a concentration of 66% (mass ratio of modified sodium silicate to total alkaline solution), forming the final alkali activator [12]. The prepared activator was aged at room temperature for 24 h prior to use to ensure thermal equilibrium.

#### 2.3.2. Exploration of the Different Mixing Methods on Geopolymer Properties

**Figure 2:**
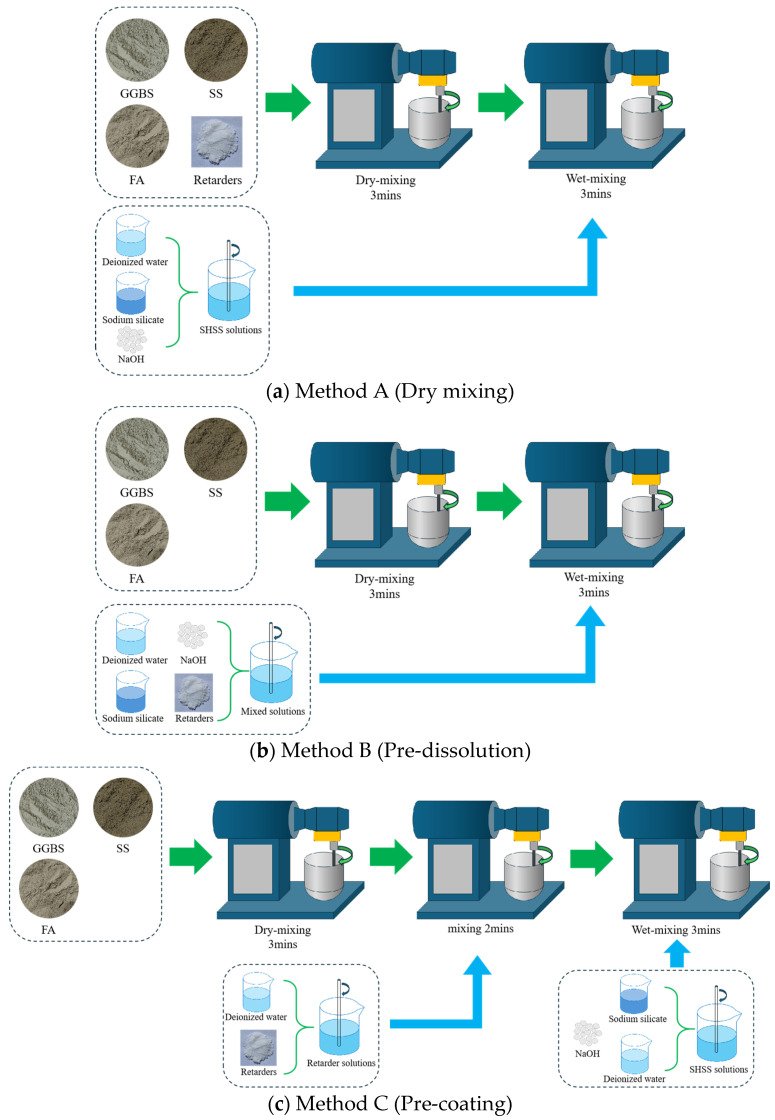
Diagram of the operating procedures for different mixing methods. (**a**) Method A. (**b**) Method B. (**c**) Method C.

Three mixing methods were performed: (A) dry mixing, (B) pre-dissolution, and (C) pre-coating. Method A involved initial dry mixing of the precursor with BaCl_2_ powder for 3 min followed by 3 min wet mixing with the alkali activator solution. In Method B, BaCl_2_ powder was first dissolved in water to form a solution, which was then blended with the alkali activator; the precursor was dry-mixed for 3 min before incorporating the blended solution through 3 min wet mixing. Method C commenced with BaCl_2_ solution preparation, followed by 3 min dry mixing of the precursor, 2 min wet mixing with the BaCl_2_ solution to ensure complete particle coating, and a final 3 min wet mixing with the alkali activator solution. The detailed mixing process is shown in Figure 2.

### 2.4. Test Methods

#### 2.4.1. Fluidity

According to the Chinese standard GB/T 2419-2005 [17], the fresh mixed paste was slowly poured into a round flow table Φ 50 × 100 × 150 mm truncated cone mold. Then, the paste was jumped 25 times within 25 ± 1 s after removing the mold. The vertical dimension of the paste was measured, and the average value was reported.

#### 2.4.2. Setting Time

The initial and final time of fresh mixed paste was determined using the Vicat apparatus in accordance with Chinese standard GB/T 1346-2011 [18]. The prepared paste was poured into a Vicat mold and flattened with a spatula. The Vicat needle was then inserted into the paste at regular intervals, and the depth was recorded to determine the setting time of the paste. The specimens were placed in a standard curing box (25 ± 1 °C, 95% R.H.) for the duration of the experiment. Triplicate tests were conducted for each mixture, and the average value was reported as the final result.

#### 2.4.3. Hydration Heat

The hydration heat of the paste was tested using calorimetry. The effect of BaCl_2_ on the hydration rate of the fresh paste within 48 h was investigated. The hydration heat of the paste was measured using an eight-channel TAM AIR isothermal calorimeter (TA Instruments, New Castle, DE, USA). Approximately 5 g of raw material was used for each test, conducted at 25 °C. The experiments were repeated, and the total heat measurement error was controlled within ±3%.

#### 2.4.4. Micro-Analysis

Geopolymer mortars of different ages were used to evaluate the evolution of hydration. After the specified age of curing, the samples were impregnated with isopropyl alcohol to stop the hydration reaction and subsequently dried in a vacuum drying oven at 40 °C for 24 h. The dried samples were ground into powder for XRD measurements. The XRD test was performed using a German Bruker D8 Advance X-ray diffractometer (Bruker AXS GmbH, Karlsruhe, German) with Cu_K-beta radiation at 40 kV and 40 mA. The test involved scanning an angular range from 5° to 70° with a step interval of 0.2° and a scanning rate of 4°/min.

The hardened geopolymer samples, prepared with BaCl_2_ precursor mass fractions of 0% and 3.0%, were characterized using a ZEISS Sigma 300 scanning electron microscope (SEM) (Carl Zeiss AG, Oberkochen, Germany) at an accelerating voltage of 20 kV. Approximately 50 mg of specimens at different curing ages were immersed in ethanol three times for 48 h each and dried. The samples were sputter-coated with gold to enhance conductivity, and their microstructures were examined by SEM. Additionally, the elemental distribution on the sample surfaces was analyzed using an Oxford Ultim Max 100 energy-dispersive spectrometer (EDS) (Oxford Instruments, Abingdon, UK).

## 3. Results and Discussion

### 3.1. Mix Proportion Optimization

#### 3.1.1. Effect of Steel Slag Content on Setting Time

**Figure 3:**
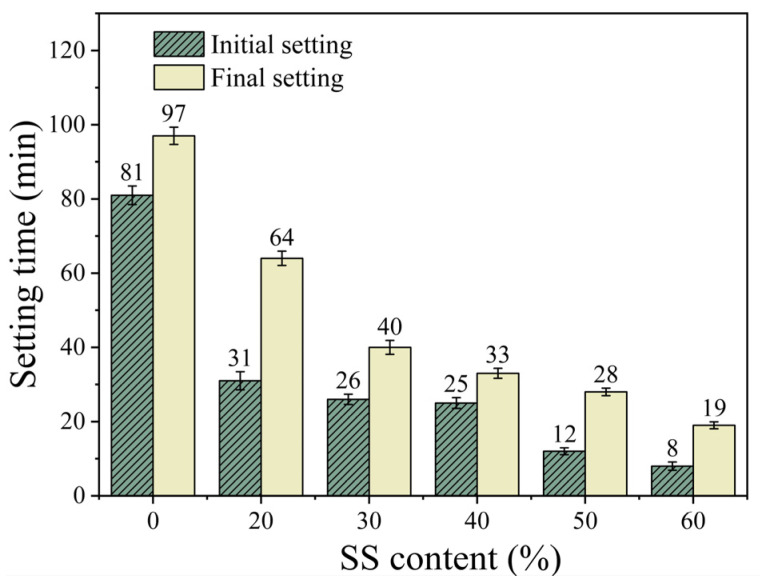
The effect of SS content on setting time.

Figure 3 illustrates the relationship between the steel slag (SS) content and the setting time of the SS-GGBS-FA ternary geopolymer mortar. When the SS content is 20%, the initial and final setting times of S20GF11 are 31 and 64 min, respectively, which are reduced by 61.7% and 34.0% compared with S0GF11. As the SS content increases to 60%, the initial and final setting times decrease to 8 and 19 min, representing reductions of 90.1% and 80.4% compared with S0GF11. This indicates that the SS content significantly affects the setting time of the ternary geopolymer, with a decrease in setting time as the SS content increases. This result contradicts the findings of Song et al. [9]. The underlying reason, as mentioned earlier, is that the alkalinity of steel slag is a crucial factor influencing its setting time. According to Table 1, the CaO content in steel slag is 40.45%, which is higher than that in mineral powder (39.37%) and fly ash (10.79%). The high CaO content increases the alkalinity and accelerates the hydration reaction [19,20,21], thereby shortening the setting time. In contrast, the steel slag used by Song was of low alkalinity. Notably, when the SS content is 40%, the initial setting time of S40GF11 is 25 min, while the final setting occurs only 8 min after initial setting, suggesting that a 40% SS content may be a key proportion. Therefore, 40% SS content was selected as the mix ratio for subsequent experiments.

#### 3.1.2. Effect of Steel Slag Content on Compressive Strength

**Figure 4:**
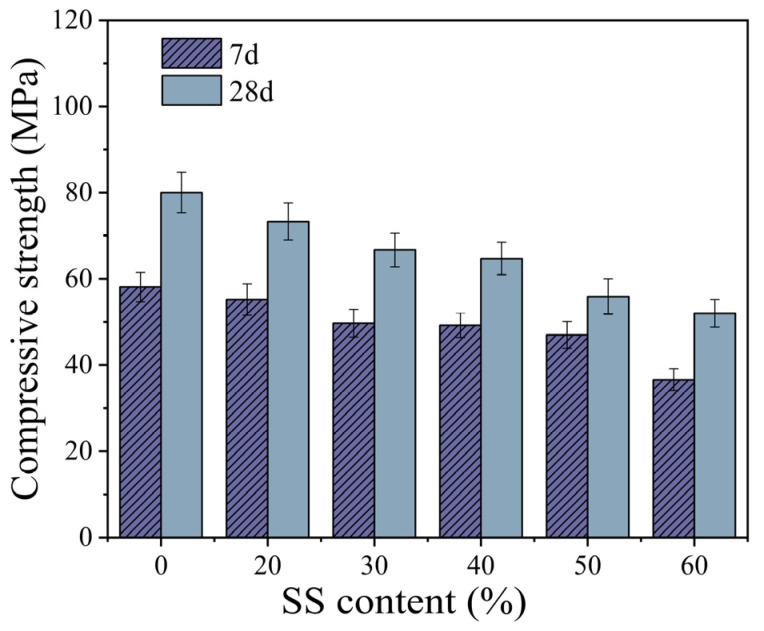
The effect of SS content on compressive strength.

As shown in Figure 4, the compressive strength of the TGM decreased with increasing steel slag (SS) content. Within the SS content range of 0–30%, the 7-day and 28-day compressive strengths of the samples decreased by 14.5% and 16.6%, respectively. When the SS content reached 60%, the 7-day and 28-day compressive strengths were reduced by 37.0% and 35.0%, respectively, compared with the control group. Additionally, as the SS content increased from 30% to 40%, the compressive strength showed only a slight decline—1.0% at 7 days and 3.0% at 28 days. However, within the range of 40–60% SS content, the 7-day and 28-day compressive strengths decreased by 25.6% and 19.6%, respectively, indicating that excessive SS incorporation significantly impairs the mechanical properties of geopolymers, particularly their early-age compressive strength. These results are consistent with findings by Song et al. [9], who reported that, as SS content increased, the compressive strength of SS–FA binary geopolymer decreased significantly, with early-age strength development being more adversely affected.

According to the above research results, it can be concluded that the setting time of TGM decreases significantly with an increasing content of high-alkalinity steel slag. It is noteworthy that, when the steel slag content exceeds 40%, the mechanical performance of TGM deteriorates markedly. To enhance steel slag utilization while maintaining acceptable mechanical properties, a steel slag content of 40% was selected as the optimal proportion for subsequent experimental investigations.

#### 3.1.3. Effect of GGBS/FA Mass Ratio on Setting Time

**Figure 5:**
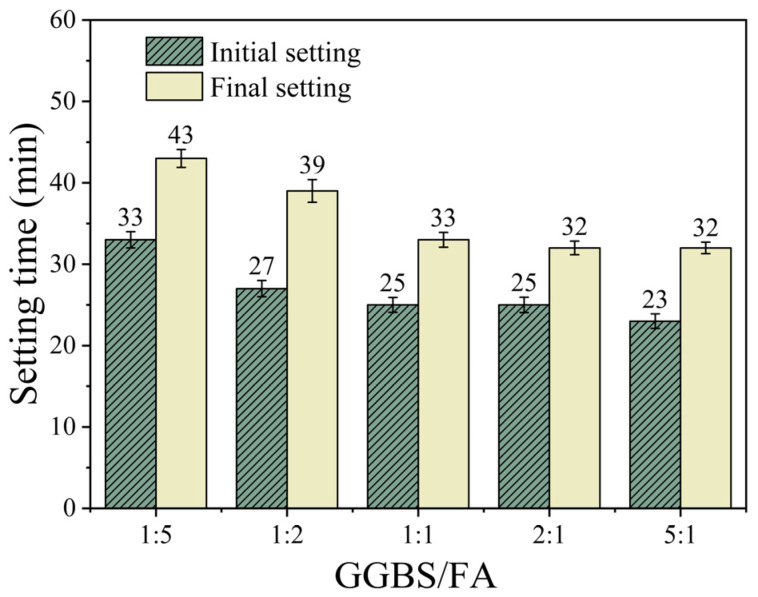
The effect of GGBS/FA mass ratio on setting time.

Figure 5 shows the effect of the GGBS/FA mass ratio on the setting time of the samples. As shown in the figure, when the SS content is fixed at 40%, the influence of the GGBS/FA mass ratio on the setting time exhibits a two-stage pattern. As the GGBS/FA ratio increases from 1:5 to 1:1, the initial setting time decreases from 33 min to 25 min (a reduction of 24.2%), and the final setting time decreases from 43 min to 33 min (a reduction of 23.3%). However, when the ratio continues to increase to 5:1, the change in setting time slows down, with the initial and final setting times decreasing by only 8.0% and 3.0%, respectively. This non-linear response pattern reveals the synergistic effect of material components: GGBS has higher hydration activity than FA and hydrates more rapidly in the early stages [22]. Moreover, due to its high CaO content, GGBS may accelerate the development of C-S-H and C-A-S-H gels, thereby accelerating the setting and hardening of the paste [19].

#### 3.1.4. Effect of GGBS/FA Mass Ratio on Compressive Strength

**Figure 6:**
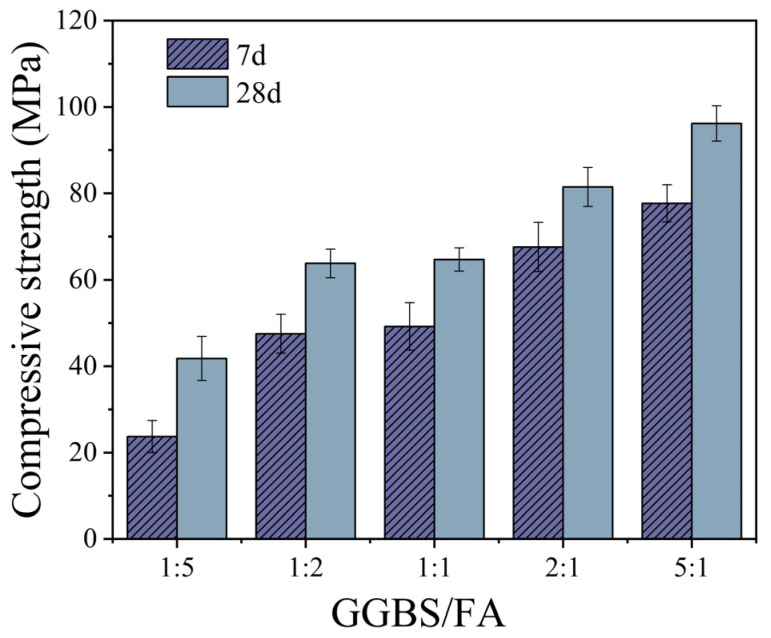
The effect of GGBS/FA mass ratio on compressive strength.

Figure 6 shows the variation in compressive strength of TGM with different GGBS/FA mass ratios. As the GGBS/FA mass ratio increases, the compressive strength of the samples improves significantly. When the GGBS/FA mass ratio increases from 1:5 to 5:1, the 7-day and 28-day compressive strengths increase by 227.8% and 130.1%, respectively. Moreover, at a GGBS/FA mass ratio of 5:1, the compressive strengths at 7 and 28 days reach 77.7 MPa and 96.2 MPa, respectively. These results indicate that the incorporation of GGBS can markedly enhance the compressive strength of ternary geopolymer systems, particularly at early ages. Numerous studies [19,23] have demonstrated that the high CaO content in GGBS promotes the formation of C-S-H and C-A-S-H gels within the matrix. The development of these gels effectively fills the micro-pores generated during the hydration process, thereby accelerating early strength development. Additionally, GGBS exhibits a nucleation effect [24]; higher GGBS contents provide additional nucleation sites, which facilitate the formation of C-S-H and C-A-S-H gels, ultimately contributing to improved compressive strength in geopolymers.

### 3.2. Effect of BaCl_2_ on Ternary Geopolymer Mortar

#### 3.2.1. Effect of BaCl_2_ Mixing Method on the Setting Time of TGM

**Figure 7:**
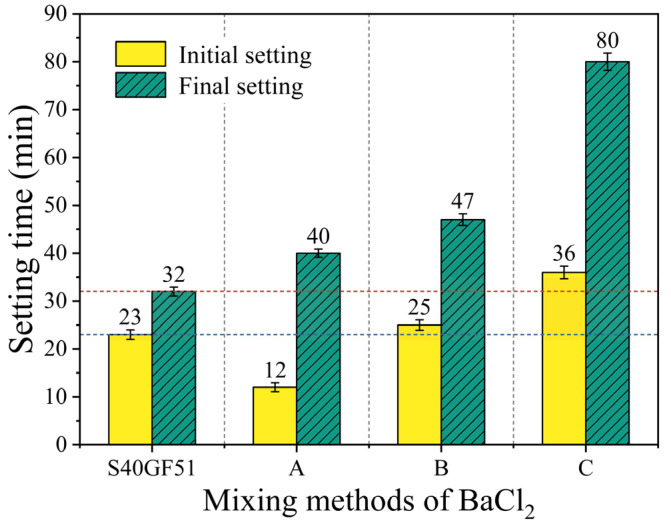
Relationship between setting time and mixing methods of BaCl_2_.

Figure 7 shows the effect of BaCl_2_ on the setting time of S40GF51 under three different mixing methods. Setting time is a key parameter for evaluating the performance of cementitious materials, with the initial setting time reflecting the early-stage setting characteristics, while the final setting time is closely related to the duration for the material to begin developing strength. Compared with S40GF51, the group with Method A (dry mixing) exhibited a significant reduction of 47.8% in the initial setting time, indicating a marked acceleration effect on the early setting process. The group with Method B (pre-dissolution) showed a slight increase of 8.7% in the initial setting time, suggesting a weak retarding effect. The group with Method C (pre-coating) exhibited a 56.5% extension in the initial setting time, demonstrating a significant retarding effect, which effectively delayed the early setting process of the paste.

In terms of final setting time, the Method A group had a final setting time of 40 min, a 25.0% increase compared to with the reference group; the Method B group had a final setting time of 47 min, an increase of 46.9%; and the Method C group had a final setting time of 80 min, an increase of 150.0%, far exceeding the increases observed in the A and B groups, thus showing excellent retarding performance. The significant retarding effect of Method C can be attributed to the thorough mixing and encapsulation of the BaCl_2_ solution with the precursor. After the addition of the alkaline activator, Ba^2+^ ions reacted with SiO_3_^2−^ ions to form BaSiO_3_ precipitates. These precipitates were encapsulated on the surface of the precursor particles, forming a barrier that hindered the intense structural disintegration of the precursor particles triggered by the alkaline activator. Related studies further support this explanation. Fan Xiaodan et al. [25] observed through scanning electron microscopy (SEM) that BaCl_2_ formed a distinct gel-like encapsulation layer on the surface of slag; Yu Qijun et al. [26] confirmed through energy dispersive X-ray spectroscopy (EDS) that this encapsulation layer was the product of the reaction between BaCl_2_ and water glass.

In conclusion, the effect of BaCl_2_ on the setting time of S40GF51 varies significantly depending on the mixing method. Method C, by forming a BaSiO_3_ precipitate layer, effectively delayed the setting process, providing a new approach for controlling the setting time of cementitious materials and offering both theoretical and experimental support for optimizing cementitious material performance.

#### 3.2.2. Effect of BaCl_2_ Dosage on the Workability of TGM

Based on the significant retarding effect of BaCl_2_ under the C mixing method, an in-depth investigation was conducted on the influence of BaCl_2_ dosage on the workability under the C mixing method.

**Figure 8:**
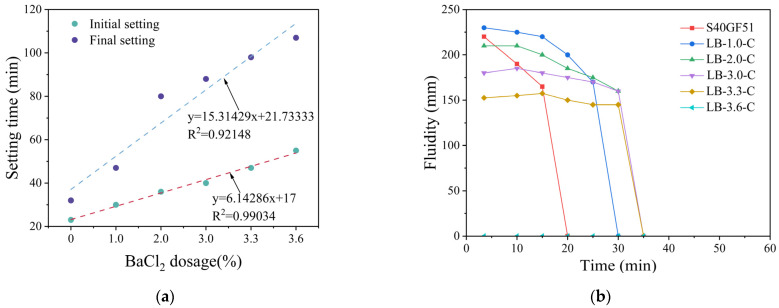
The relationship between workability and BaCl_2_ content. (**a**) Setting time. (**b**) Fluidity. Note: LB-1.0-C represents the incorporation of 1.0% BaCl_2_ into S40GF51 using the C mixing method.

The effect of BaCl_2_ dosage on the setting time of S40GF51 was observed under the C mixing method, with BaCl_2_ dosages of 1.0%, 2.0%, 3.0%, 3.3%, and 3.6%. As shown in Figure 8a, with the increase in BaCl_2_ dosage, both the initial and final setting times were significantly prolonged. Specifically, as the BaCl_2_ dosage increased from 0% to 3.6%, the initial/final setting times extended from 23 min and 32 min to 55 min and 107 min, respectively, representing an increase of 139% and 234%. This indicates that the incorporation of BaCl_2_ significantly extends the setting time of S40GF51. In this study, the relationship between the setting time of S40GF51 and the dosage of BaCl_2_ can be well fitted using a first-order polynomial expression. The coefficients of determination (R^2^) obtained from the regression analysis indicate a clear linear relationship between BaCl_2_ dosage and both the initial setting time (R^2^ = 0.99034) and the final setting time (R^2^ = 0.92148) of the TGM.

The flowability tests were conducted 3.5 min after the addition of the activator, with the results shown in Figure 8b. The maximum flowability of S40GF51 at 2.5 min was 220 mm, which gradually decreased within 10 to 20 min. As the BaCl_2_ dosage increased, the initial flowability first rose to the maximum value and then decreased to zero flowability, with the duration of flowability extended. For instance, when the BaCl_2_ dosage was 3.0%, the maximum flowability reached 185 mm at 10 min, and then gradually declined until it sharply dropped to zero between 30 and 35 min. Compared with S40GF51, the flowability retention time was extended by 15 min, and the time to reach the maximum flowability was delayed by 7.5 min.

It is noteworthy that the flowability at 3.5 min initially increased and then decreased with an increasing BaCl_2_ dosage, with the maximum value of 230 mm observed at a BaCl_2_ dosage of 1.0%. Subsequently, within the BaCl_2_ dosage range from 1.0% to 3.3%, the flowability continued to decrease, and, at a content of 3.6%, the slurry lost its flowability. In S40GF51, when a large amount of highly reactive components comes into contact with the alkaline activator solution, C-(A)-S-H gel forms within a short time, resulting in a higher dynamic yield stress of the slurry. The appropriate incorporation of BaCl_2_ can encapsulate the highly reactive particles, hindering the rapid formation of the gel, thereby reducing the dynamic yield stress of the slurry [15], which is reflected in the increased flowability. However, an excessive BaSiO_3_ encapsulation layer may lead to increased friction between particles within the slurry, reducing its flowability.

#### 3.2.3. Effect of BaCl_2_ Dosage on the Compressive Strength of TGM

**Figure 9:**
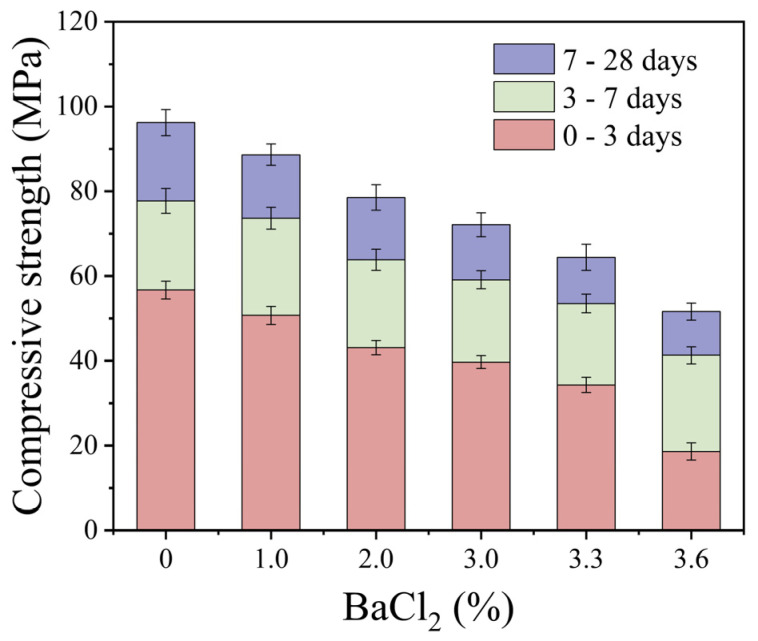
The effect of BaCl_2_ dosage on compressive strength.

Figure 9 depicts the compressive strength of S40GF51 under various curing times and different dosages of BaCl_2_. The results indicate a continuous decrease in compressive strength as the BaCl_2_ dosage increases within the range of 0% to 3.6%. Compared with the control group, the compressive strength of the 3.6% BaCl_2_ group decreased by 67.2%, 46.8%, and 46.3% at 3, 7, and 28 days, respectively. This suggests that BaCl_2_ has a more pronounced impact on early-age strength. BaCl_2_ reacts with Na_2_SiO_3_ in the alkali activator to form BaSiO_3_ precipitates, which adsorb onto the surface of precursor particles, thereby inhibiting their dissolution and obstructing the formation of hydration gels in the initial stages of the reaction. However, even at 28 days, the 3.6% BaCl_2_ group still exhibited a 46.3% reduction in compressive strength. This continued strength loss can be attributed to the partial consumption of alkali activators by BaCl_2_, which hinders the dissolution and breakdown of the precursors, subsequently reducing the generation of hydration products. Although the incorporation of BaCl_2_ may lead to strength reduction in geopolymers, the 28-day compressive strength of the 3.0% BaCl_2_ group still reached 72.1 MPa.

### 3.3. Heat of Hydration Analysis

**Figure 10:**
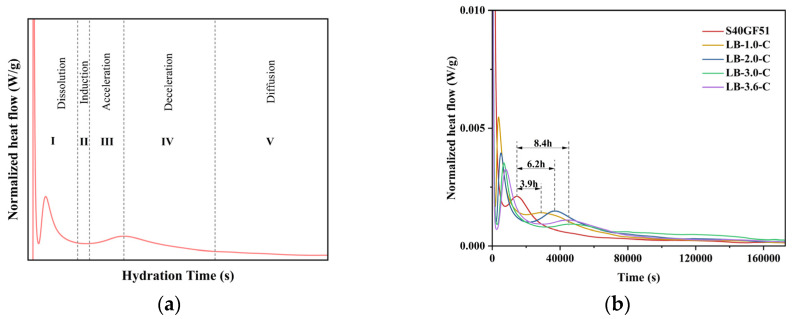

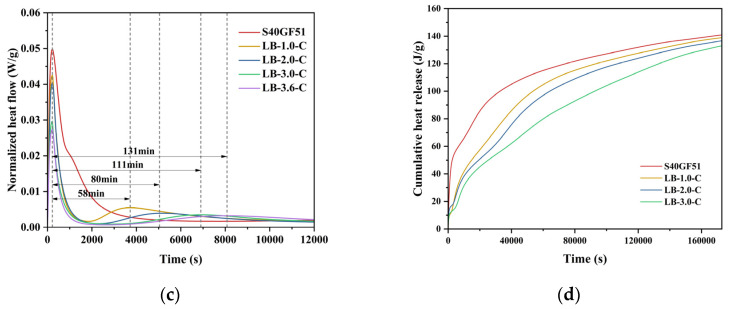
Hydration characteristics of samples with BaCl_2_. (**a**) Hydration process model. (**b**) Hydration heat release rate. (**c**) Exothermic diagram of heat flow. (**d**) Cumulative heat release.

The hydration exothermic characteristics of S40GF51 under different BaCl_2_ dosages are shown in Figure 10. The experimental results are similar to the five stages of the hydration process of alkali-activated slag cement [27] (dissolution, induction, acceleration, declaration, and diffusion stages, as shown in Figure 10a). During the hydration process, the S40GF51 group exhibited two peaks in the calorimetry. The first peak appeared at 220 s (dissolution stage), while the second peak occurred at 14,560 s (acceleration stage). It is noteworthy that the addition of BaCl_2_ resulted in two calorimetry peaks during the hydration dissolution stage, with the heat release at the first peak being higher than that at the second peak. He et al. [28] studied alkali-activated slag–fly ash–silica fume cement and found that two calorimetry peaks also appeared in the dissolution stage, with the second peak being higher than the first. They pointed out that, when the precursor comes into contact with the alkali activator, Ca^2+^ ions begin to dissolve, initiating the first calorimetric peak. Subsequently, the Ca^2+^ ions react with the Si^4+^ and Al^3+^ ions dissolved from the precursor, leading to the formation of the second calorimetric peak. However, in their study, the calcium content in the precursor was relatively low, which explains why the second peak was higher than the first. In contrast, the present ternary system is a high-calcium system, and the single peak observed in the hydration dissolution stage of S40GF51 was primarily due to the heat released from the dissolution of CaO. With the incorporation of BaCl_2_, the BaSiO_3_ encapsulation layer hindered the dissolution and erosion process of the alkali activator with active components like CaO, leading to a reduction in the peak value of the first calorimetry peak. Meanwhile, the depolymerization–polymerization reactions between the precursor and alkali activator continued slowly, and the dissolved Ca^2+^ ions reacted with the Si^4+^ and Al^3+^ ions in the precursor, forming a second relatively lower calorimetric peak in the dissolution stage. Further studies revealed that, as the BaCl_2_ dosage increased (1%, 2%, 3%, and 3.6%), the first calorimetric peak value during the hydration dissolution stage gradually decreased. Additionally, the second calorimetric peak was delayed relative to the first peak by 58 min, 80 min, 111 min, and 131 min, respectively. Meanwhile, compared with S40GF51, the acceleration stage was delayed by 3.9 h, 6.2 h, 8.4 h, and 8.4 h, respectively.

Figure 10d shows the cumulative heat release during hydration. The total heat release increased and then decreased with the increase in BaCl_2_ dosage, as the incorporation of BaCl_2_ consumed the alkali activator to varying degrees. After 48 h, the total heat release of S40GF51, LB-1.0-C, LB-2.0-C, and LB-3.0-C slurries were 140.9 J/g, 138.9 J/g, 136.6 J/g, and 133.0 J/g, respectively. Compared with the control group, the total heat release reduction rates for BaCl_2_-incorporated slurries were 1.4%, 3.1%, and 5.6%, respectively. It can be observed that the 2.0% BaCl_2_ dosage had almost no impact on the hydration of the geopolymer, while the 3.0% BaCl_2_ dosage affected the overall hydration of the geopolymer to some extent.

### 3.4. Microstructural Analysis

#### 3.4.1. XRD Analysis

**Figure 11:**
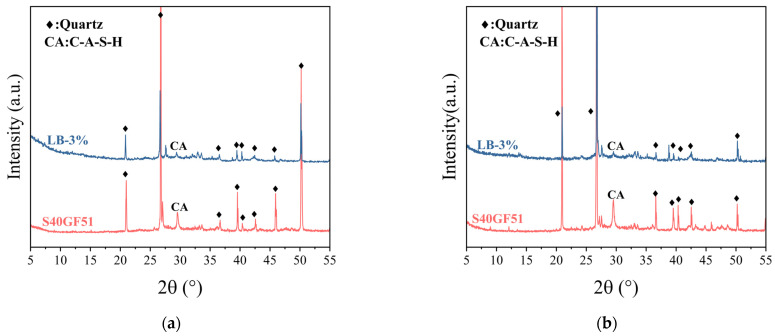
XRD pattern of hardened samples at initial setting and 1 day: (**a**) initial setting; (**b**) 1 day.

Figure 11 displays the X-ray diffraction (XRD) patterns of the samples after the initial setting and 1-day curing revealed the reaction characteristics of different geopolymers. The gel in high-calcium system geopolymers is mainly amorphous C-A-S-H gel [29]. In an alkaline environment, the Ca-O bond is more likely to break and combine with Si^4+^ to form C-S-H gel, while the presence of Al^3+^ promotes the transformation of C-S-H into C-A-S-H [30]. Comparing the LB-3.0-C sample with S40GF51, a diffraction peak corresponding to C-A-S-H gel (PDF # 00-020-0452) was observed at 2θ = 29.5° for both samples. However, the peak intensity for LB-3.0-C was weaker, indicating that the formation of C-A-S-H gel was inhibited. In S40GF51, dissolved Ca^2+^ or Al^3+^ (H_4_AlO_4_) rapidly reacts with [SiO_2_(OH)_2_]^2−^, forming a large amount of C-A-S-H gel in a short time, accelerating the setting of the slurry [31]. The incorporation of BaCl_2_ solution leads to the reaction of Ba^2+^ with SiO_3_^2−^ in the alkali activator to form BaSiO_3_ precipitates, which consumes the alkali activator, slows down the hydration reaction, and reduces the formation of C-A-S-H gel. However, due to the relatively low amount of BaCl_2_ added, no significant BaSiO_3_ diffraction peak was observed in the XRD patterns. It has been reported that there is a synergistic effect in the hydration of steel slag and slag [32], which can produce C-A-S-H gel during hydration, contributing to the improvement of the compressive strength of geopolymers [33]. After one day of curing, no new diffraction peaks appeared in the LB-3.0-C sample, except for quartz and C-A-S-H, indicating that the incorporation of barium chloride does not change the main types of hydration products in S40GF51.

#### 3.4.2. SEM-EDS Analysis

**Figure 12:**
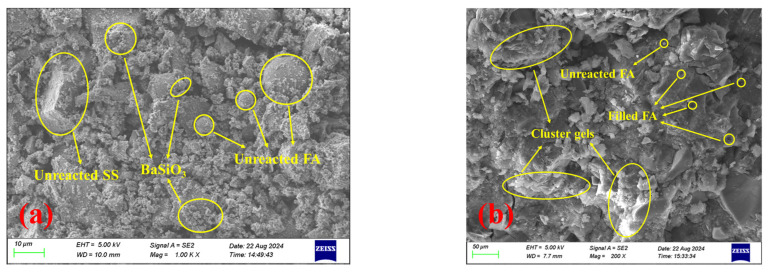

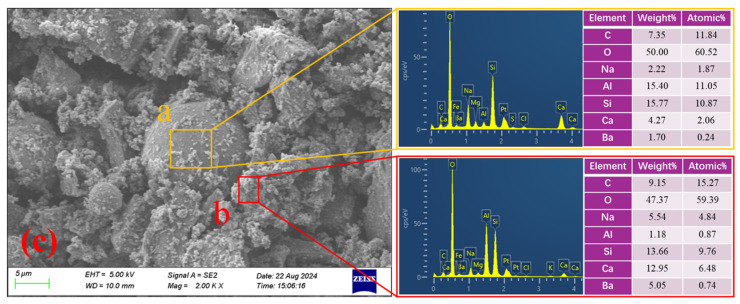
SEM images of LB-3.0-C and S40GF51 samples at initial setting. (**a**) LB-3.0-C. (**b**) S40GF51. (**c**) EDS image of LB-3.0-C.

To investigate the retarding mechanism of BaCl_2_ on S40GF51 under the C mixing method, SEM/EDS analysis was conducted on the initial setting samples of LB-3.0-C and S40GF51. As shown in Figure 12, the microstructure of S40GF51 is dense, containing only a small amount of unreacted FA, indicating a high degree of alkali activation. Additionally, large amounts of needle-like, cluster-like, and amorphous gel products cover and bind residual particles, forming a continuous block gel that reduces porosity and enhances mechanical strength. These gels are primarily composed of C-S-H or C-A-S-H gels formed by the reaction of Ca^2+^ ions released from CaO in SS and GGBS with the alkali activator. In contrast, the microstructure of the initial setting sample from the LB-3.0-C group is loose and heterogeneous, with a significant amount of unreacted or partially dissolved FA and SS particles, suggesting a lower degree of alkali activation. Furthermore, the precursor particles are enveloped by flocculent gel-like substances suspected to be BaSiO_3_ precipitates. As shown in Figure 12c, spectrum a has a low Ca content and relatively high and similar amounts of Al and Si, indicating that this particle is unreacted FA. Additionally, the spectrum shows a lack of Ba, which is likely sourced from BaSiO_3_ precipitates on the particle surface. In spectrum b, there is a higher content of Ba, Si, and O, indicating the presence of BaSiO_3_ precipitates. The EDS images further confirm the presence of unreacted FA particles and the encapsulation of these particles by BaSiO_3_ precipitates, indicating that the formation of BaSiO_3_ hinders the dissolution and hydration of the precursor particles.

### 3.5. Retarding Mechanism of BaCl_2_ Under Method C

**Figure 13:**
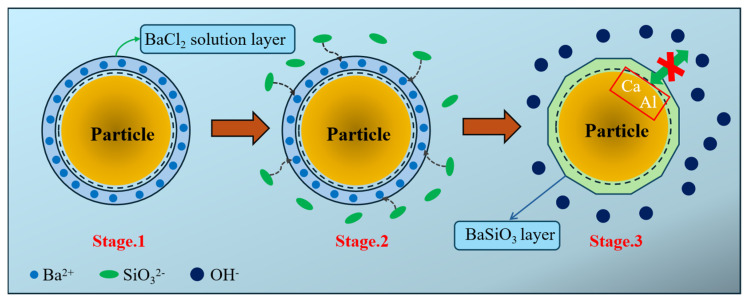
The retarding mechanism model of BaCl_2_ on ternary geopolymer.

Based on the research findings on the effects of BaCl_2_ on the setting time and flowability of ternary geopolymers under the C incorporation method, combined with the studies on the phase composition, microstructure, and hydration exotherm of geopolymers, a comprehensive model for the retarding mechanism of BaCl_2_ on ternary geopolymers under the C incorporation method is proposed, as shown in Figure 13.

Mechanism analysis indicates that, during the pre-encapsulation stage (Stage 1), the C mixing method involves pre-mixing the BaCl_2_ solution with precursor particles, which leads to the attachment of the BaCl_2_ solution to the surface of the precursor particles, forming a BaCl_2_ solution coating. In the subsequent alkali activation stage (Stage 2), Ba^2+^ ions from the BaCl_2_ solution coating preferentially react with the silicate ions (SiO_3_^2−^) released from the alkali activator, generating an insoluble BaSiO_3_ precipitate layer (Stage 3). This layer, with a thickness ranging from a nano- to a micron-scale, covers the surface of the precursor particles. The BaSiO_3_ precipitate layer plays a dual role in regulating the setting behavior. First, as a physical barrier, it effectively reduces the contact area between the alkali activator and precursor particles, delaying the alkali solution’s disintegration effect on the precursor silica–alumina network. Second, the Ba^2+^ ions consume the alkaline SiO_3_^2−^ ions in the alkali activator, lowering the liquid phase alkalinity of the system, which in turn inhibits the depolymerization–polymerization reaction rate of the precursor particles in the highly alkaline environment.

It is important to note that all experiments in this study were conducted in a standard laboratory environment with a controlled temperature of 20 ± 2 °C. The experimental results indicate that, under the C mixing method, the retarding effect of BaCl_2_ on TGM remains stable and effective within this temperature range.

## 4. Conclusions

This study investigated the effects of steel slag content and the GGBS/FA mass ratio on the setting time and compressive strength of SS-GGBS-FA ternary geopolymer mortar. Based on the optimized mix design (S40GF51), the influence of the BaCl_2_ retarder mixing method and dosage on the workability and compressive strength of S40GF51 was further explored. The retardation mechanism of BaCl_2_ was elucidated at the microstructural level through XRD, SEM, and EDS analyses, and a corresponding retarding mechanism model was established. Based on the experimental results, the main conclusions are as follows:

1. Incorporating high-alkalinity steel slag significantly accelerates the setting of TGM but reduces its compressive strength. At 60% SS content, the initial and final setting times are reduced to 8 and 19 min, respectively. Moreover, excessive steel slag leads to a rapid decline in compressive strength.
2. Increasing the GGBS/FA mass ratio shortens the setting time of the TGM while significantly improving its compressive strength. As the GGBS/FA ratio increases from 1:5 to 5:1, the 7-day and 28-day compressive strengths increase by 227.8% and 130.1%, respectively.
3. The BaCl_2_ mixing method and dosage strongly influence the setting behavior of TGM. Method C (pre-coating) shows the best retardation performance by forming a BaSiO_3_ layer around precursor particles, effectively delaying the setting time. Under Method C, both initial and final setting times increase markedly with higher BaCl_2_ dosages.
4. XRD, SEM, and EDS analyses indicate that under the C mixing method, BaCl_2_ forms a BaSiO_3_ precipitate layer that encapsulates the precursor particles. This encapsulation delays the early-stage hydration reactions and reduces the formation of early hydration products.
5. The BaSiO_3_ precipitate layer formed under Method C retards the reaction through a dual mechanism: it physically hinders the interaction between the alkali activator and precursor particles, and chemically reduces the alkalinity by consuming SiO_3_^2−^ ions, thus delaying the depolymerization–polymerization process.

## Figures and Tables

**Table 1:** Chemical composition of raw materials (wt%).

Raw Material	SiO_2_	CaO	Al_2_O_3_	Fe_2_O_3_	MgO	MnO	SO_3_	TiO_2_	K_2_O	P_2_O_5_
GGBS	30.1	39.37	15.5	0.43	6.26	0.19	2.18	0.734	0.328	-
FA	41.8	10.79	23.54	4.36	1.97	0.08	3.27	1.28	1.94	0.52
SS	11.7	40.45	3.51	27.03	6.14	2.09	0.27	0.691	0.036	1.92

**Table 2:** Design of mixing proportions of geopolymer.

Sample	Raw Material/g			Activator		W/B
	SS	GGBS	FA	Modulus	Alkali Content/%	
S0GF11	0	250	250	1.5	6	0.33
S20GF11	100	200	200			
S30GF11	150	175	175			
S40GF11	200	150	150			
S50GF11	250	125	125			
S60GF11	300	100	100			
S40GF15	200	50	250			
S40GF12		100	200			
S40GF21		200	100			
S40GF51		250	50			

Note: Sample S40GF51, S40 indicates that the steel slag content is 40%. GF51 indicates that the mass ratio of GGBS/FA is 5:1.

## Data Availability

The original contributions presented in this study are included in the article. Further inquiries can be directed to the corresponding author.
